# The role of rice husk biochar addition in anaerobic digestion for sweet sorghum under high loading condition

**DOI:** 10.1016/j.btre.2020.e00515

**Published:** 2020-08-03

**Authors:** Haiyuan Ma, Yong Hu, Takuro Kobayashi, Kai-Qin Xu

**Affiliations:** a Center for Material Cycles and Waste Management Research, National Institute for Environmental Studies, Tsukuba 305-8506, Japan; b Fujian Ospring Technology Development Co., Ltd., No. 22 Jinrong North Road Cangshan District, Fuzhou 350000, China

**Keywords:** Sweet sorghum, Anaerobic digestion, Rice husk biochar, Alkalinity, Direct electron, Transfer

## Abstract

Biochar is a carbon rich product made from the biomass pyrolysis process. Recently, biochar addition in anaerobic digestion processes has attracted attention for its possible functions to act as pH stabilizing agent, microbial carrier, and interspecies electron transfer. In this study, the effects of rice husk biochar addition in sorghum anaerobic digestion were investigated in batch tests. Under high F/M (food to microorganism) ratio conditions, obvious pH decreases and volatile fatty acids (VFA) accumulation was observed. Addition of 15 g/L biochar was found effective to increase the sorghum maximum methane production rate by 25 % and shorten the lag phase time by 44 %. Further biochar concentration increase showed little effects. Alkalinity increase brought by biochar addition contributed to the performance enhancement. Moreover, the promotive effects of biochar addition on degradation of acetic acid, propionic and butyric acid were not obvious under neutral pH conditions.

## Highlights

• Rick husk biochar addition mitigated the pH decrease during high load sorghum AD.
• 20 g/L biochar addition reduced the peak total VFA by 375 mg/L at F/M ratio of 1.2.
• Maximum methane production rate was increased by 25 % with 15 g/L biochar addition.
• Lag phase time was decreased by 45 % with 15 g/L biochar addition.

## 1 Introduction

Large areas of the arable land in Northeast Japan were seriously damaged after the Great East Japan Earthquake and the Fukushima Daiichi nuclear disaster. Organic matters and fertility deficiency of the soil after decontamination were the main problems faced by these areas. For fertility recovery and efficiently use of sterile land, a feasible solution is to cultivate energy crops which could serve as green manure as well as energy resources. Among lots of crops, sorghum is characterized with high biomass yield and desirable chemical composition to be converted to bioenergy [1]. The rhizome remained in the land during one harvest of sorghum could be up to 20 t/ha, which brings enough organic matters into the soil. The harvested sorghum could be further used as the substrate of bioenergy production [2].

Anaerobic digestion is one of the most efficient treatment approaches for different kinds of waste biomass such as sewage sludge, livestock manure, food waste and agricultural waste [3,4]. Nowadays, the anaerobic digestion of energy crops has been widely applied in Europe. It is reported that as high as 80–90 % of the biogas plants in Germany used energy crop alone or with other waste biomass as substrate [5]. Biochar is generated as a byproduct of waste biomass pyrolysis, which is featured with high proportion of carbon and porous structure and has been widely used as a soil amendment in agro-ecosystems [6]. In Japan, about 2 million tons of rice husk are produced annually in the rice threshing process [7]. Part of the rice husk is used in composting but a big fraction was still not properly used. Open burning of rice husk could cause serious air pollution and has been prohibited by environmental regulations in Japan [8]. Moreover, crystalline silica produced during burning has been determined as a carcinogen. Gasification technology of rice husk could effectively prevent the air pollution by process control and recover electricity, heat. Additionally, biochar was produced during the pyrolysis at the same time. Recently, the combined system of pyrolysis and anaerobic digestion has been attracting more and more attention [9].In the rural area, the produced biochar could be directly added in the anaerobic digestion reactor to help stabilize the process as well as to improve the methane production efficiency [6]. In the situation of mono-substrate treatment of high lignocellulose biomass, the combined system of pyrolysis and anaerobic digestion provides a better strategy to achieve further economic benefits. The merits of biochar addition in anaerobic digestion are mainly reported to be the adsorption of organic compounds or heavy metals [10,11], the increase of buffering potential [12], the potential of direct interspecies electron transfer (DIET) [13], and to act as the microbial carriers to increase the microbe concentration [14].

High lignocellulose biomass such as sorghum is formed of recalcitrant structure consisting cellulose, hemicellulose and lignin, which become an obstacle of hydrolysis process during anaerobic digestion [15]. Different efforts have been made to improve the digestibility of lignocellulose biomass such as pretreatments, co-digestion with other nitrogen rich substrates. Most of the pretreatment methods are still lack of economic feasibility due to the high energy cost [15]. In most cases, the volumetric biogas production rate is a main factor affecting the economic feasibility of biomass anaerobic digestion [16]. However, under excessively high volumetric organic loadings, unbalance between acidogenesis and methanogenesis induced VFA accumulation could lead to serious inhibition of biomass digestion process [17]. This easily happens at digestion system of substrate lack of buffering capacity. Energy crops are typical substrate with low alkalinity due to the high ratio of C/N [18]. Organic loading must be decreased, or excess alkalinity should be supplied to avoid further inhibition. Previous studies have confirmed the validity of biochar addition during anaerobic digestion process of easily hydrolyzed substrates such as food waste and organic fraction of municipal solid waste (OFMSW). Enhancements of VFA degradation rate and methane production rate were observed in the digestion of these substrates [19].

However, the application of biochar addition on high lignocellulose biomass is rarely reported. In this study, the effects of F/M ratios on sorghum anaerobic digestion, and the role of biochar addition under high F/M ratio were investigated by batch experiments.

## 2 Materials and methods

### 2.1 Substrate and inoculated sludge

Harvested sorghum was firstly dried at 50 ℃ for 2 days and then crushed to the size of less than 1 cm before the experiments. The characteristics of the prepared sorghum are shown in Table 1. The organic elements C, H, O, N were analyzed via a fundamental measurement service of National Institute for Environmental Studies by using an element analyzer Flash EA 1112 (Thermo Electron Corporation, US). The TS, VS and COD were analyzed according to the standard methods [20]. The cellulose (ADF-ADL), hemi-cellulose (NDF-ADF) and lignin (ADL) contents of the sorghum were analyzed in the fractions of NDF (neutral detergent fiber), ADF (acid detergent fiber) and ADL (acid detergent lignin) were analyzed according to a previous report [21]. The inoculated sludge was taken from a lab-scale anaerobic CSTR (continuous stirred tank reactor) reactor treating sorghum at mesophilic condition. The total solids (TS) of the reactor was around 30 g/L and the organic loading rate (OLR) of the reactor was 2 g−COD/L/d.

**Table 1:** Characteristics of dried sorghum used in this study (based on raw material weight).

Component	Value
C (%wt)	43.59 ± 0.25
H (%wt)	5.66 ± 0.08
O (%wt)	35.76
N (%wt)	0.78 ± 0.09
TS (%)	91.8 ± 0.3
VS (%)	85.8 ± 0.23
Cellulose (%wt)	37.9
Hemi-cellulose (%wt)	23.3
Lignin (%wt)	4.9

TS: total solids.

VS: volatile solids.

### 2.2 Biochar

The biochar was produced by rice husk pyrolysis (thermal gasification) under 600 ℃ and the main characteristics of the biochar used are shown in Table 2. The organic elements C, H, O, N was analyzed by using an element analyzer Flash EA 1112 (Thermo Electron Corporation, US). The total alkalinity was quantified by reaction with HCl and subsequent back titration described in a previous study [22]. Exchangeable K, Mg and Ca were extracted with 1 M NH_4_OAc (pH 7) and determined by inductivity coupled plasma optical emission spectrometer (ICP-OES).

**Table 2:** characteristics of the rice husk biochar used in this study.

Parameter	Value
VS (%wt)	39.39 ± 0.07
ash (%wt)	59.54 ± 0.04
C (%wt)	33.93 ± 0.58
N (%wt)	0.19 ± 0.01
H (%wt)	0.78 ± 0.05
Total alkalinity (cmol/kg)	43.96 ± 0.66
Exchangeable K (cmol/kg)	16.5
Exchangeable Mg (cmol/kg)	2.2
Exchangeable Ca (cmol/kg)	7.2

### 2.3 Batch experiments

Batch experiments were conducted to investigate the roles of biochar addition in high loading sorghum fermentation. Different F/M ratios were tested to simulate different loading conditions and the effects of biochar addition were investigated under high loading conditions.

Firstly, to evaluate the effects of substrate induced inhibition on sorghum anaerobic digestion, batch experiments were conducted at F/M ratios of 0.1, 0.3, 0.6, 0.9, 1.2, 1.8, 2.4. For each test, 50 mL of seed sludge, corresponding amount of sorghum at set F/M ratio based on VS concentrations, and 30 mL of deoxygenated water were added in serum vials. Then the serum vials were sealed with rubber stoppers and aluminum caps. The head space of the serum vials was purged with pure nitrogen gas to obtain anaerobic condition. The vials were set at a shaker (120 rpm) and cultivated in a thermostat (35℃). The gas production volume was determined by syringe and gas components of N_2_, CH_4_ and CO_2_ were analyzed by a gas chromatography (GC-8A, Shimadzu) equipped with TCD detector. The GC column used was a stainless packed column (Porapak Q80/100, Agilent). Liquid samples were taken for pH and VFA analysis. pH was analyzed by a compact pH meter (AS-pH-22, HORIBA) and VFA was analyzed by a gas chromatography (GC-2014, Shimadzu) equipped with FID detector. A capillary column (DB-WAXetr, 30 m, 0.53 mm, 0.5 μm, Agilent) was used for VFA detection. Each test was conducted in duplicate.

After previous experiments, a F/M ratio high enough to induce inhibition was determined. Different biochar addition concentrations were selected to verify its effects on sorghum anaerobic digestion. The experiments were conducted following the same process described above except for extra biochar addition. The biochar addition concentration was set at 0, 5, 10, 15, 20 g/L based on the seed sludge volume. The main VFAs detected during the high F/M ratio tests were then used directly as substrate to investigate the role of biochar addition in degradation of these VFAs by batch tests. For each vial, 50 mL of seed sludge, and 30 mL of VFA solutions were added and operated as the same procedure described above. The pH of the VFA solutions were adjusted to 7.0–7.5 by adding 1 M NaOH solutions. The concentration of VFAs in the vials were set at 2000 mg-COD/L. Biogas production, pH and VFA concentration variation were monitored until the biogas production almost stopped.

### 2.4 Methane production parameters calculation

Gompertz equation was widely used in the evaluation of biomass anaerobic digestion for its capability to determine the methane production potential, methane production rate and lag phase time before the rapid methane production [23]. In this study, the relationship between methane production and cultivate time was simulated by using modified Gompertz equation:

(1) $$P=P_{0}\cdot \exp\cdot \left\{-\exp\left[\frac{R_{\max}\cdot e}{P_{0}}\cdot \left(t_{0}-t\right)+1\right]\right\}$$

Where P represents the methane production volume (NmL), P_0_ represents the methane production potential (NmL), R_max_ represents the maximum methane production rate (NmL/gVS_added_/d), t_0_ represents the lag phase time (days) and t represents the reaction time (days).

## 3 Results and discussion

### 3.1 Effects of F/M ratios on sorghum anaerobic digestion

**Fig. 1:**
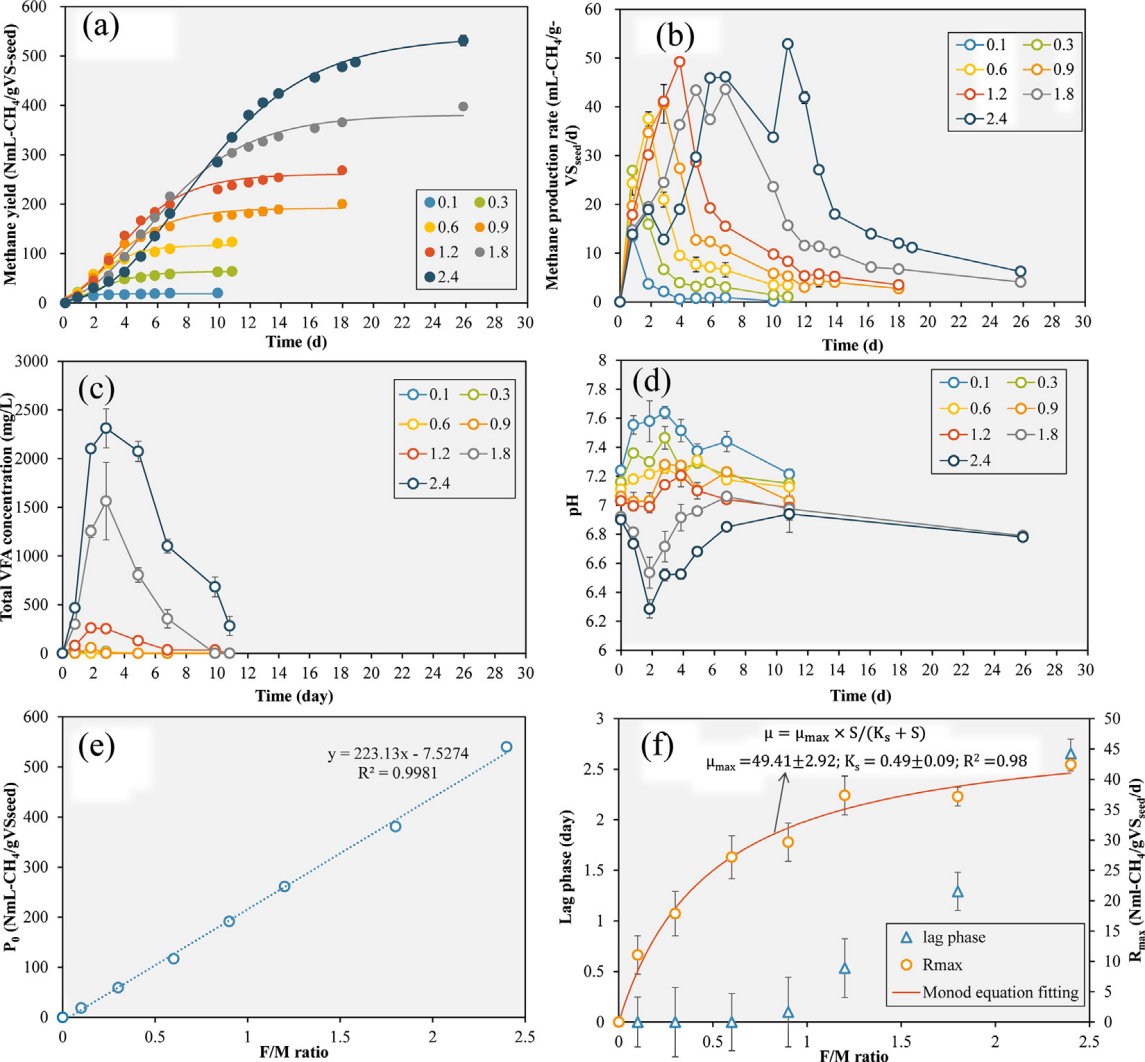
The methane production performance from low to high organic loadings: methane yield and Gompertz equation fitting curves (a), methane production rate (b), total VFA concentration (c), pH variation (e) during F/M ratio batch tests, methane production potential at different F/M ratios (e), lag phase, R_max_ calculated by Gompertz equation and Monod equation fitting curve (f).

To determine the effects of substrate loading on sorghum anaerobic digestion, the methane production, pH variation and VFA accumulation during sorghum anaerobic digestion were monitored in a series of batch tests at different F/M ratios (Fig. 1). The relationship between methane yield and incubation time under all the F/M ratio conditions fitted the Gompertz model well with R^2^ > 0.93. With the increase of F/M ratio from 0.1 to 2.4, the methane production potential increased linearly from 18.5 to 540 NmL-CH_4_/gVS/d (Fig. 1e). It could also be observed that maximum methane production rate increased significantly when F/M ratio was increased from 0.1 to 1.2 and kept relatively stable between F/M ratio of 1.2 and 2.4 (Fig. 1f). The relationship between maximum specific methane production rate and F/M ratio fitted the Monod equation well with R^2^ of 0.98. The maximum methane production rate R_m_ and half velocity constant K_s_ were calculated to be 49.41 ± 2.92 NmL-CH_4_/gVS_seed_/d and 0.49 ± 0.09 respectively. Under higher F/M ratios, after the incubation start, methane yields along with incubation time showed a typical lag phase, followed with an almost linearly increase of methane production and a stable stage in the final stage. The lag phase time started to increase when F/M ratio was set higher than 0.9, which was almost 0 at F/M ratio of 0.1 to 0.6 but increased to 0.10, 0.53, 1.29, 2.66 days at F/M ratio of 0.9, 1.2, 1.8 and 2.4, respectively (Fig. 1f).

VFA accumulation is a common phenomenon under high organic loading rate during the anaerobic digestion process. In this study, obvious VFA accumulation was observed when F/M ratio was raised to over 0.9. The total VFA concentration reached 260 mg/L on day 1.8 when F/M ratio was set at 0.9. The total VFA even accumulated to over 1500 mg/L and 2000 mg/L temporarily when F/M ratios were at 1.8 and 2.4. The VFA accumulation indicated the unbalance between sorghum hydrolysis, acidogenesis and methanogenesis. The methanogenesis process becomes the rate-limit step under high organic loading pressure and vigorous acidogenesis.

When the F/M ratio was set as high as 1.8 and 2.4, the VFA accumulation exceeded the system buffering capacity, and pH decreased from around 6.9 at the beginning to 6.5 and 6.3 on day 1.83, respectively. The methanogenesis rate at this time was suppressed due to the deviation of pH from the optimal range. It could be observed that from day 0.80 to day 2.82, although VFA concentration was adequate, the methane production rate under F/M ratio of 1.8 and 2.4 was obviously lower than the groups with lower F/M ratios due to pH inhibition on methanogenesis (Fig. 1b). Subsequently, with the VFA consumption proceeding, the pH recovered to the neutral range and the methane production rate also increased to its maximum value. With further F/M ratio increase, further pH decrease below 6.3 could be speculated and could cause serious inhibition or even completely cease of methanogenesis [23].

### 3.2 Effects of biochar addition on sorghum anaerobic digestion

**Fig. 2:**
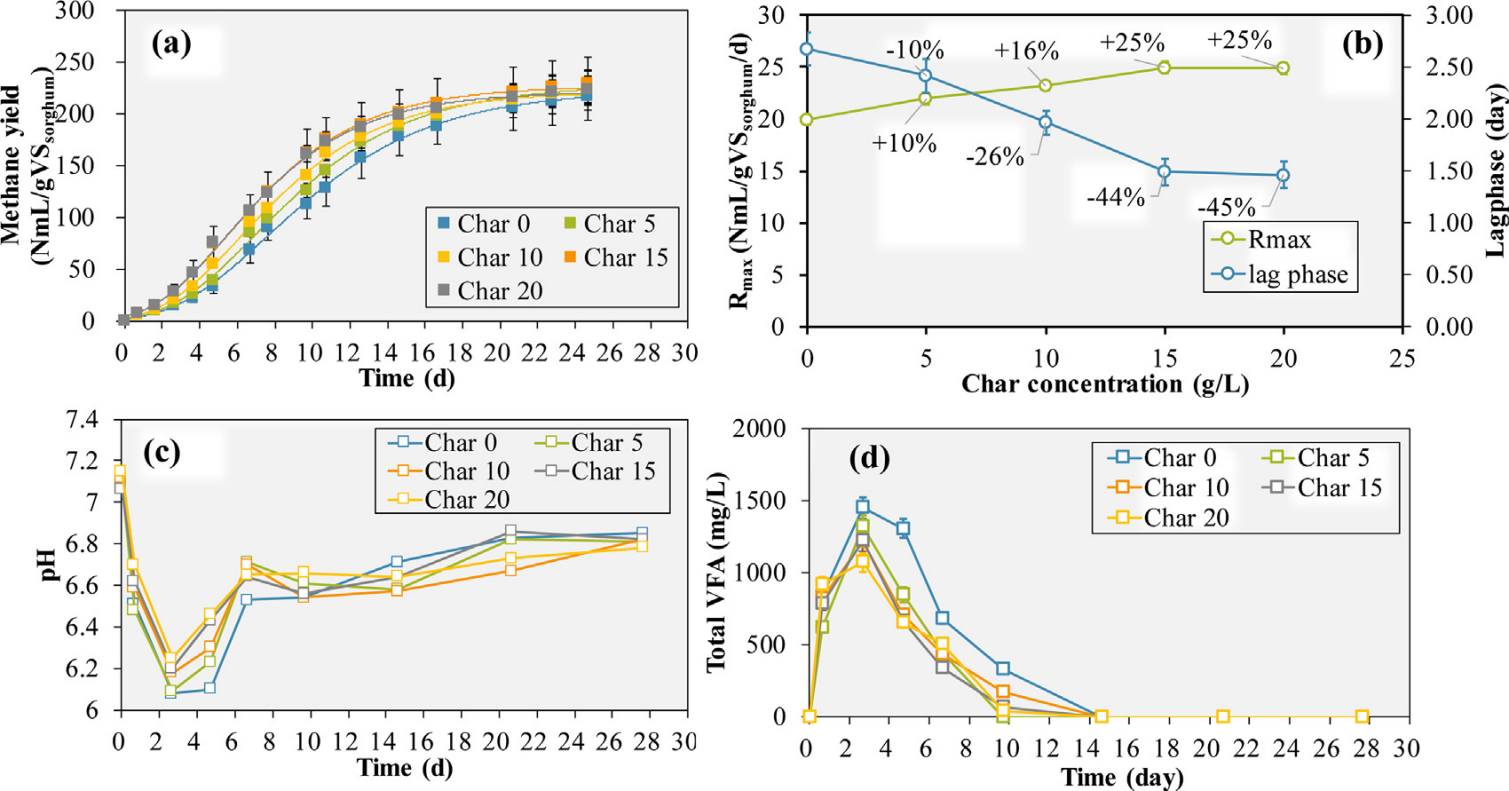
The methane yield and Gompertz equation fitting curves (a), R_max_ and lag phase calculated by Gompertz equation (b), pH (c) and total VFA concentration (d) variation during batch tests under different biochar addition concentrations.

Biochar is reported to be capable of adjusting pH due to its abundant negative organic function groups or inorganic carbonate formed during pyrolysis [9] and was applied in this study to investigate its effects on sweet sorghum anaerobic digestion. In section 3.1, higher substrate loading induced VFA accumulation and pH decrease were observed. Especially, when F/M ratio was higher than 1.2, the methane production rate decreased due to unfavorable pH conditions. Therefore, effects of different biochar concentrations (0, 5, 10, 15 and 20 g/L) were tested at F/M ratio of 1.2, and methane production performance was monitored (Fig. 2).

According to the organic element analysis, the sorghum used in this study could be expressed as CH_1.56_O_0.62_N_0.02_, and an equation (eq. (2)) could be established to estimate the methane production [24].

(2) $$\text{C}{\text{H}}_{1.56}{\text{O}}_{0.62}{\text{N}}_{0.02}+0.335{\text{H}}_{2}\text{O}\to {0.533\text{C}\text{H}}_{4}+0.448{\text{C}\text{O}}_{2}+0.02{\text{N}\text{H}}_{4}^{+}+0.02{\text{H}\text{C}\text{O}}_{3}^{-}$$

By using eq. (4), the methane production potential from complete sorghum degradation is around 504 N mL/gVS. Due to the actual methane production potential simulated by Gompertz equation, methane production potential for different groups was almost the same (221–228 NmL/gVS_sorghum_), which indicated the sorghum VS degradability to be around 45 %. The VS reduction of lignocellulose biomass such as sorghum was relatively low because of the recalcitrant structure and lignin content. Nevertheless, the methane yield and VS reduction could also vary as a result of different between substrate and inoculum. Generally, methane yield of 200–330 NmL/gVS with VS reduction rate of around 50 % from sorghum was reported in the previous studies [25,26].

Although no obvious differences of total methane production volume were observed between the groups with or without biochar addition, the methane production started faster in the groups with biochar addition. The relationship between methane yield and culture time fitted well with the Gompertz equation and was used to evaluate the effects of biochar addition on methane production. With the biochar addition concentration increased from 0 to 15 g/L, the maximum methane production rate R_max_ increased from 19.9–25.0 Nml-CH_4_/gVS_sorghum_/d (25 % increased) while the lag phase decreased from 2.67 to 1.49 days (44 % decreased). However, further increase of biochar concentration from 15 to 20 g/L showed almost no effect on both R_max_ and lag phase time (Fig. 2(b)).

Besides, pH variation was mitigated due to the addition of biochar. At the start of the experiments, the pH in different vials was in the range between 7.09 and 7.15. With the reaction proceed, the pH of all groups dropped to the lowest on day 2.67. However, pH values of the vials with addition of 5, 10, 15, 20 g/L biochar were 6.09, 6.18, 6.20, and 6.25 on day 2.67 respectively, higher than the pH of 6.08 in the vial without biochar addition. The optimal pH range of methanogenic microbes is around 6.6–7.6 and pH lower than 6.3 was reported to inhibit the methanogenesis rate [23]. The rice husk biochar used in this study showed a total alkalinity of 43.96 ± 0.66 cmol/kg, which is equal to 439.6 mg-CaCO_3_/L of alkalinity when rice husk biochar was added at concentration of 20 g/L. Biochar addition significantly increased the system alkalinity and kept the pH variation close to the neutral pH range even under high organic loading conditions. Similar phenomenon of CH_4_ production rate enhancement and pH decrease alleviation was also observed in the anaerobic digestion of easy-acidification substrate by vermicompost biochar addition [12].

It should be noticed that alkalinity of different biochars varied in a wide range according to the feed stock and pyrolysis conditions. The alkalinity of biochars was reported to be correlated with acid soluble base cations and could be higher than 150 cmol/kg and 250 cmol/kg respectively for biochars made from hard wood and mixed wood [22]. Therefore, the biochar addition concentration in the purpose of alkalinity supply in anaerobic digestion should be discussed case by case.

**Fig. 3:**
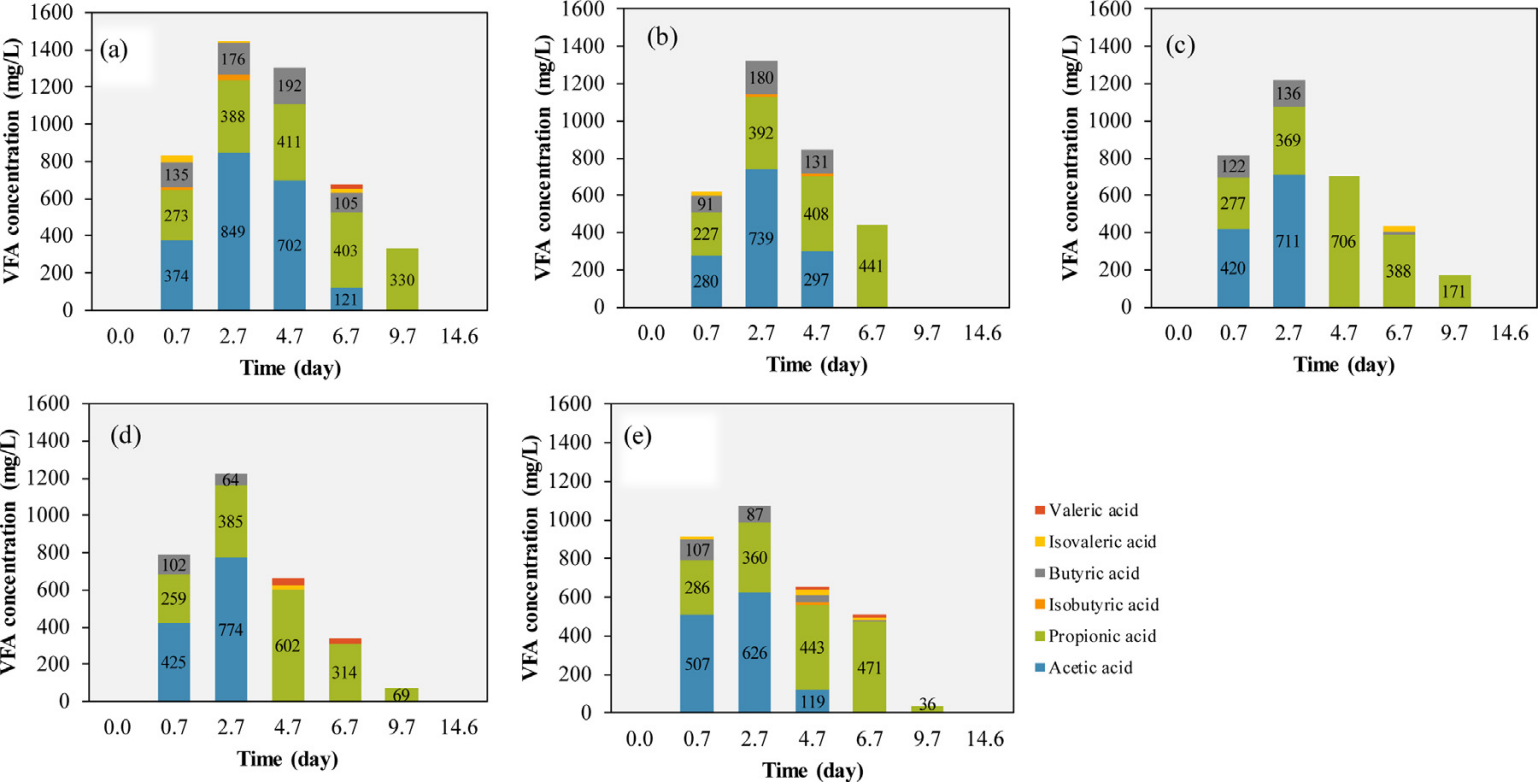
The VFA accumulation during the batch tests under different concentration of biochar: 0 (a), 5 (b), 10 (c), 15 (d), 20 g/L (e) biochar addition.

Additionally, biochar addition also alleviated the VFA accumulation during the experiments. Compared with the vials without biochar addition, the VFA conversion rate was enhanced and the total VFA concentration decreased from 1448 to 1073 mg/L on day 2.7 with 20 g/L biochar addition. The main VFAs accumulated during the experiments were acetic acid, propionic acid and butyric acid, which accounted for over 90 % of the total VFA (Fig. 3). In all groups, the VFA concentration increased in the first 2.7 days. On day 2.7, VFA concentration in all vials reached the highest level with over 50 % of acetic acid. After that, the acetic acid started to decrease but the propionic acid continuous to accumulate in the following day 2.7−4.7. The butyric acid was depleted at almost the same time with the acetic acid. In this experiment condition, acetic acid accounted for a higher proportion when the total VFA concentration reached the highest level, but the propionic acid seemed to be more difficult to be consumed during the experiment.

### 3.3 Effects of the biochar addition on different VFA degradation

Besides as a buffering agent, conductive materials such as biochar was also reported to be effective to promote butyric and propionic acid degradation by direct interspecies electron transfer (DIET) functions between acidogenesis and methanogenesis microbes to relieve the VFA accumulation [19,27]. But the effects of DIET under high organic loadings could not be estimated due to the pH increase brought by biochar addition.

**Fig. 4:**
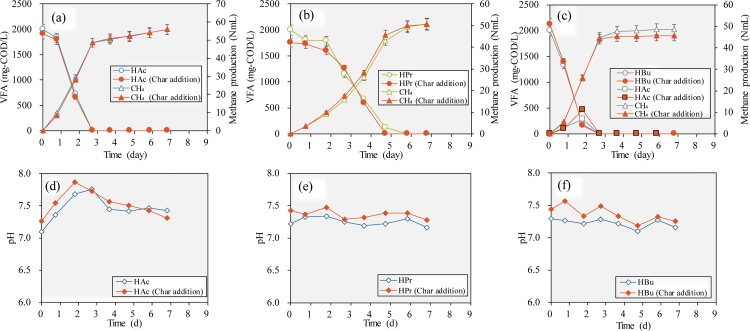
The effect of biochar addition on acetic acid (a, d), propionic acid (b, e), and butyric acid (c,f) degradation.

During anaerobic digestion of sorghum, acetic acid, propionic acid, and butyric acid were the main VFAs detected to accumulate under high organic loadings. Effects of biochar addition on the conversion of these VFAs were tested in batch tests (Fig. 4). Unlike the experiments directly used sorghum as substrate, the pH at the beginning of these test was adjusted to be between 7.0–7.5 and stayed stable between the range of 7.1–7.9 for acetic acid group, 7.2–7.5 for propionic acid group and 7.1–7.6 for butyric acid group. The addition of 15 g/L biochar increased the pH of the vials around 0.2 during the batch tests but still maintained the pH in the optimal range for methane production.

Methane production from acetic acid degradation was the fastest in the beginning as methanogens could directly convert acetic acid to methane and CO_2_. The methane production almost finished in 2.7 days and acetic acid concentration decreased to 0 at the same time. Almost no difference was observed between the groups with and without biochar addition. Propionic and butyric acids are commonly considered difficult to be oxidized because of thermodynamically unfavourability without the syntrophic hydrogen consuming of methanogens [28]. Moreover, microbe concentrations of propionic and butyric oxidizing bacteria are much lower compared with methanogens. The methane production from propionic acid degradation was the slowest among the three kinds of VFAs and finished on day 5.82. The methane production rate from the biochar addition group was found only a slightly higher than the control group. During the propionic acid degradation, no acetic acid was detected, which means a very fast conversion from acetic acid to methane and propionic acid oxidation was the rate limit step. In the occasion of butyric acid, the finish time of methane production was the same with that of acetic acid degradation in 2.7 days. Because degradation rate of butyric acid was much higher compared with propionic acid, acetic acid was detected as an intermediate product from the start to day 2.7. Especially, on day 1.8, acetic acid concentration in biochar addition group reached 465 mg-COD/L, which was 180 mg-COD/L higher than that of control group. This phenomenon indicated that biochar addition promoted the conversion of propionic to acetic acid. However, the methane production rate in the propionic acid group showed no significant difference with or without biochar addition.

The role of biochar as stabilizing agent for anaerobic digestion has been widely reported [9]. The pH decrease under high organic loading conditions during the substrate acidogenesis was mitigated by biochar addition in this study. A shorter lag phase and a higher maximum methane production rates were observed mainly due to pH buffering ability of biochar as well as the potential direct interspecies electron transfer between fermentative bacteria and methanogens [9]. Mathematical modeling indicated that DIET rate could be 8.6 folds higher than hydrogen transfer [13], and the conductive materials could help to transfer the electron to electrotrophic methanogens which made the degradation of propionic or butyric acid possible under high hydrogen partial pressures. However, this also depends on the existence of specific microbes capable of electric syntropy such as *Geobacter* species and *Methanosaeta*, which need to be cultivated after an acclimation period of biochar addition. In the condition of anaerobic digestion system under proper pH condition and with healthy methanogens community, promotive effects of further biochar addition on VFA conversion were limited.

## 4 Conclusions

In this study, the role of rice husk biochar addition on sorghum anaerobic digestion under high loading conditions was investigated and key findings could be summarized as follows:

• Lag phase increased significantly along with F/M ratio increase and F/M ratio higher than 1.2 induced obvious pH decrease and VFA accumulation.
• The biochar addition of 15 g/L was found effective to increase the sorghum maximum methane production rate by 25 % and shorten the lag phase time by 44 %. Further biochar concentration increase showed little effects.
• The promotive effects of biochar addition on degradation of acetic acid, propionic and butyric acid were limited under neutral pH conditions.

## CRediT authorship contribution statement

**Haiyuan Ma:** Writing - original draft, Writing - review & editing, Investigation. **Yong Hu:** Methodology. **Takuro Kobayashi:** Funding acquisition, Supervision. **Kai-Qin Xu:** Supervision, Writing - review & editing.

## Declaration of Competing Interest

We declare that we have no financial and personal relationships with other people or organizations that can inappropriately influence our work, there is no professional or other personal interest of any nature or kind in any product, service and/or company that could be construed as influencing the position presented in, or the review of, the manuscript entitled.
